# Evaluation of Real-Life Investigational Use of Enoximone in Asthma, the Third Step in Drug Repurposing: A Preliminary Report

**DOI:** 10.1155/2021/7456208

**Published:** 2021-11-01

**Authors:** Jan Beute, Pieter Boermans, Alex KleinJan

**Affiliations:** ^1^Almere, Netherlands; ^2^Red Cross Hospital, Beverwijk, Netherlands; ^3^Department of Pulmonary Medicine, Erasmus University Medical Center (Erasmus MC), Dr. Molewaterplein 50, 3015 GE Rotterdam, Netherlands

## Abstract

**Background:**

The population of uncontrolled asthma patients represents a large therapeutic burden. The PDE3-inhibitor enoximone is a strong and quick bronchodilator and is known to successfully treat life-threatening bronchial asthma (status asthmaticus). Translational mice models showed anti-inflammatory effects when PDE3 was targeted.

**Methods:**

Here, we investigated the effectiveness of PDE3-inhibitor enoximone as oral treatment for chronic asthma in a real-life off-label setting. Investigational use of PDE3-inhibitor enoximone: 51 outpatients (age 18–77) with chronic asthma were followed using off-label personalized low doses of the PDE3-inhibitor enoximone. Duration of treatment was 2–8 years.

**Results:**

Four groups could be distinguished as follows: The first group includes patients who use enoximone as an add-on, because it helps them in maintaining a better general wellbeing; they still use their traditional medication (*n* = 5). The second group consists of patients who use enoximone and were able to phase down their traditional medication without deterioration of their asthma symptoms (*n* = 11). The third group comprises patients who were able to discontinue their traditional medication and use only enoximone without deterioration of their asthma symptoms (*n* = 24). The last one has patients who, after having used enoximone for some time, saw their symptoms disappear and now use no medication at all, not even enoximone (*n* = 11). All patients reported improvement or at least alleviation of their asthma symptoms. All patients reported a better quality of life and greater drug compliance.

**Conclusion:**

The evaluation shows that PDE3-inhibitor enoximone is a viable alternative for or addition to current asthma therapeutics, as both add-on and stand-alone, considerably reducing the use of LABAs/SABAs/ICS, with no or negligible side effects. Additional studies are advisable.

## 1. Introduction

Asthma is an inflammatory obstructive airway disease [1, 2]. Causes for developing asthma are to be found in an interplay of environmental factors and the degree of exposure to those factors, combined with a multitude of genetic variations that become apparent in a wide range of immune cells and structural cells. In the last two decades, improvement regarding asthma treatment has been marginal; inhaled corticosteroids (ICS) are still the main therapy [3, 4]. Treatment with biologicals shows a reduction of ICS, but they are only useful when patients present a very specific distinct endotype and are not suitable in acute exacerbations. Other ‘new' therapies are merely variations on ICS, or, for children, on drugs that were allowed for adult asthma patients, such as beta-2-mimetics, anticholinergic agents, magnesium sulphate, and aminophylline (clinicaltrials.gov). In extreme bronchoconstriction, all of these are of little or no avail [5–7]. In the past decades, the literature has hinted at the therapeutic potential of phosphodiesterase-3(-PDE3)-inhibitors in asthma [7–9]. The most well-known nonspecific PDE3-inhibitor is theophylline, having a limited dosage efficacy, narrow therapeutic window, and a weak bronchodilator effect [8]. Another PDE3-inhibitor, enoximone, was developed for treatment of heart failure in the 1980s; it increases cyclic adenosine monophosphate (cAMP) and ionized calcium in the myocyte, resulting in increased myocardial contractility. In vascular wall smooth muscle cells, enoximone causes an increase in cAMP and in cyclic guanosine monophosphate (cGMP), resulting in relaxation and subsequent vasodilatation; the same applies to bronchial smooth muscle cells, causing bronchodilation [10, 11]. In heart failure, a disease with a very complex pathophysiology, PDE3-inhibitors were banned because they led to faster heart deterioration and increased mortality (in high doses up to 2400 mg/day) [12]. However, since the 90s, enoximone, in ten times lower doses, is standard care in ICU and perioperatively, to enhance cardiac and pulmonary function [10, 13]. Based on the idea of bronchodilation, enoximone has been used successfully in 8 cases of status asthmaticus after all other treatments according to asthma guidelines had failed [5, 6]. This in turn led to preoperative treatment, in small doses, for asthma patients, who are known to face higher risk of pulmonary complications during and after surgery [14].

Translational preclinical studies in house dust mite-driven asthma models showed that targeting PDE3 reduced airway inflammation and epithelial mast cell activation [15, 16]. Human granulocyte activation with Platelet Activating Factor (PAF) or f-Met-Leu-Phe (fMLP) induces CD11b expression, which could be truncated by PDE3-and/or PDE4-inhibition [16, 17]. Reduction of eosinophilic inflammation resulted in reduced exposure to harmful eosinophil peroxidase (EPO) and reactive oxygen species (ROS). Exposed to EPO and ROS, the mucosa develops mucosal leakage, tissue edema, complement activation, and immune activation, as often seen in severe asthmatics [18–20]. PDE3-inhibition reduces granulocyte-macrophage colony-stimulating factor (GM–CSF)– production of the epithelial cell cultures, supports tight junction protein expression, and improves mucosal barrier function [21]. In vivo, it shows anti-inflammatory effects by reduced serum Tumor Necrosis Factor-alpha (TNF-*α*) levels [22, 23].

PDE3-inhibition provides multiple effects:

- Improvement of mucosal barrier function in cAMP dependent changes of the cytoskeleton and tight junctions, effectuating a decreased sensitivity to allergy related stimuli [24]
- Direct smooth muscle relaxation
- Reduction of IgE-induced inflammation

Recent clinical trials include biologicals that target merely one specific inflammatory mediator; the most important outcome variables are the reduction in inhaled ICS and exacerbations. Guidelines from EMA and FDA propose a fixed dose for clinical trials, but real life represents a different situation. Real-life asthma investigation primarily focuses on the patient and on patient-reported outcome and thus offers opportunity for personalized medication ((long-term) asthma patients are quite capable of assessing their own dosage). Studies for EMA or FDA drug registration only allow patients with strict compliance to the study protocol; without strict compliance, patients will be regarded as study dropouts. Unfortunately, this practice leads to omission of potentially relevant study results.

Drug repurposing is an interesting field of reinvestigating well-known drugs for new indications [25]. Roughly, the subjected drug should meet the following criteria:

- Evidence from literature regarding possible effects [5]
- Translational studies showing beneficial effects [13]
- Small pilot studies (real-life experiences/investigation) that should be performed, showing beneficial effects [20]
- Single and/or multiple escalation dose finding studies that should be performed, showing correlation between pharmacokinetics and pharmacodynamics

This paper reports the results of long-term real-life investigational use of orally administered enoximone, using a personalized dose, for maintenance treatment of (chronic) asthma, showing a decrease in asthma symptoms, an improvement in quality of life, a substantial reduction in ICS/LABAs/SABAs, and a high patient compliance to the drug.

## 2. Case Series

### 2.1. Route to Investigational Use

The literature reports side effects of both high doses of enoximone (2400 mg/day) and relatively low doses of enoximone (150 mg/day) in the treatment of heart failure [12, 26, 27]. High doses meant detrimental side effects; low doses meant no therapeutic effect and no or limited adverse side effects. After discontinuation of the use of catecholamines, PDE3-inhibitors, including enoximone, have been standard of care over the past 20+ years, perioperatively and in the ICU, in heart failure and pulmonary obstructive disease. The immediate and positive effect of a bolus of enoximone in eight near fatal cases of status asthmaticus launched a new train of thought, earmarking enoximone as a treatment for (chronic) asthma [5]. Consequently, 25 mg enoximone (Perfan ®, Carinopharm GmbH, Elze, Germany) (a dose indicated to be within safety limits [27]), was administered preoperatively to asthma patients, given that these patients are known to run a higher risk of pulmonary complications during and after surgery [14]. The results were positive, whereupon some of the patients asked for continuation of treatment after hospital discharge, as they felt that their current therapy lacked sufficient result or produced unwanted side effects. Due to successful treatment in hospital and the domino effect by word of mouth, over the past 8 years, a number of 63 patients found their way to low-dose enoximone for the treatment of asthma, including 12 minors [28].

### 2.2. Inclusion and Exclusion Criteria

No explicit exclusion criteria were used, neither in age, nor in morbidity, as the literature indicates that the extreme low dosage used in this investigation is not hazardous to any disease or disorder. Pregnant patients were advised to give birth first before starting treatment. Patients who turned out to be suffering from pulmonary aspergillosis due to frequent/long-term steroid use were advised to treat the fungal infection first.

### 2.3. Enoximone Use by Patients

An initial daily dose was determined (see Safety and Dosage), the first one administered under auspices of a physician; follow-up took place within a week (or sooner, if necessary), concerning asthma control, wellbeing, and side effects. A questionnaire was presented, addressing asthma symptoms, used medication/side effects, exercise capacity, wellbeing, and quality of life up to the moment of first interview; the same questionnaires were handed out approximately two months later in order to evaluate the differences. During the first month, the patients were contacted and evaluated every week. Over time, contacts were less frequent (but still ongoing) and eventually mostly coincided with the request for a new prescription. As this paper describes investigational use of enoximone in asthma, no blood values or other invasive measurements were involved.

Enoximone is currently only available in a liquid formulation. This concerns an intravenous solution containing ethanol and propylene glycol; for oral intake, patients mix this liquid with a drink, such as a soda, coffee, tea, or fruit juice. One patient noticed that enoximone, taken with milk or yogurt, seemed to require a longer exposure time, which suggests that somehow a chemical reaction occurs with (probably) a milk protein which possibly delays the pharmacological effect; this led to discouraging milk and yogurt as an administering aid. Water is an option but does not mask the somewhat bitter taste of the solution, which can be unpleasant for some people.

### 2.4. Safety and Dosage

Enoximone for asthma is a new indication that has not yet been described in medical literature up to now, so an algorithm for optimal dosage was formed, based on the above-mentioned literature, the experiences with the status-asthmaticus patients in the Emergency Room [5], and the preoperative treatment of severe asthma patients, and on extensive research on mice [16]. The premise was to search for the lowest effective dose, the frequency of administration ideally being 1 dd, or, in the absence of complaints, as needed. Eventually, the algorithm showed an average dose of 0,0625–0,125 mg/kg bodyweight, which translates to a personalized dosage of 5–10 mg dd for adults. A maximum dose of 20 mg dd is advised, or, if the frequency is lower, a maximum of 25 mg per administration (150 mg/day is considered within safety limits [27]).

Patients were asked to confer in case they felt the need to (temporarily) increase/reduce their dose.

### 2.5. Ethical Agreements

CCMO and METC permission do not apply since this is an investigational use report. Health Care Inspection in The Netherlands was contacted in 2014 to report our intention and to ensure that enoximone treatment for asthma would follow the rules for off-label administration. From 2018 to 2020, the therapy was audited; no irregularities were observed and clearance to proceed was obtained. Use is according to Dutch law and follows the principles of the Declaration of Helsinki. Informed consent was asked and obtained from all patients.

## 3. Results

### 3.1. Four Different Case Groups

**Figure 1:**
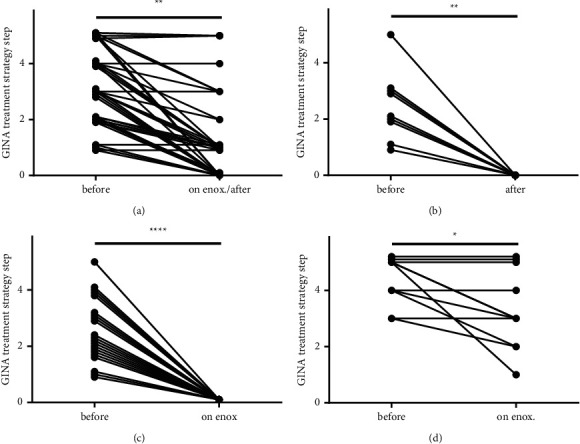
Asthma patients were able to phase down asthma medication when enoximone was taken (ICS use reduction according to GINA guidelines). Asthma patients were treated at least 3 months with enoximone (*n* = 41; enoximone 15–105 mg/week (in 1–7 dosages)). (a) Asthmatic patients (*n* = 41) using or having used enoximone (on enox./after). (b) No traditional asthma medication was needed any more (*n* = 9). (c) Only enoximonetherapy with incidental SABA/LABA (*n* = 21). (d) Enoximone together with ICS (*n* = 11) (Wilcoxon matched-pairs signed rank test ^*∗*^ = *p* < 0.05; ^*∗∗*^ = *p* < 0.01; ^*∗∗∗*^ = *p* < 0.001) (enox. = enoximone).

Four groups emerged within the treated patients (*n* = 51; age 18–77 years) (Table 1 and Figure 1):

- Group 1: patients who use enoximone as an add-on, along with their traditional medication (*n* = 5)
- Group 2: patients who use enoximone and were able to reduce their traditional medication (*n* = 11)
- Group 3: patients who were able to discontinue their traditional medication and use only enoximone (*n* = 24)
- Group 4: patients who, after having used enoximone for some time, saw their symptoms disappear and now use no medication at all, not even enoximone (*n* = 11)

The most important observation regarding clinical advance and follow-up is that *all* patients experienced improvement in wellbeing, even if they could not phase down their traditional medication; patient-reported outcome varied from more stamina, more air, and easier breathing, to an increase in quality of life, including being able to (better) perform everyday activities such as sports, social functions, and work. As for specific asthma symptoms, results such as less coughing/wheezing, less fatigue, better endurance, less sick days, and less hospitalization were reported. A secondary effect was that several of the patients noticed a decrease in their asthma-related comorbidities such as hay fever, rhinitis, eczema, and allergies (group 1, Table 1).

Another relevant observation was that 11 patients (group 2, Table 1) saw that the use of their traditional asthma medications, especially steroids, drastically reduced and that another 35 patients (groups 3 and 4, Table 1) were able to discontinue these altogether. Unexpectedly, the patients who were able to discontinue their traditional medication (within 10 weeks (median); (immediately 1 year (range)) and use only enoximone form the largest group (*n* = 24 (group 3, Table 1). 11 patients (group 4, Table 1) were able to discontinue not only their traditional medication, but also the enoximone, without return of complaints.

Retrospective analysis of the use of topical, inhaled corticosteroid (ICS) by asthmatic patients, treated with enoximone, shows that the majority of the asthmatic patients could phase down their ICS medication (*p* < 0.01Figure 1(a); ICS medication was measured according to the GINA treatment strategy steps classification). Subdivided in treatment groups: no asthma medication was needed (*n* = 9) (*p* < 0.01) after a period of enoximone add-on therapy (1¾ *y*; ½ - 5y median *y*; range y) (Figure 1(b)), only enoximone therapy with incidentally SABA/LABA (*n* = 21) (*p* < 0.0001) (Figure 1(c)) and enoximone together with ICS (*n* = 11) (*p* < 0.05) (Figure 1(d)). None of the patients showed progressive disease or hospitalization after starting enoximone treatment. Patients turned out to be highly motivated to continue enoximone therapy because of its immediate effect; this notably favours good compliance [29–31].  Table 2 summarizes patient group characteristics including the baselines age, sex, duration of treatment, and the dosage.

### 3.2. Adverse Events/Beneficial Effects

In our total patient group of 51 treated with low-dose enoximone, only 3 patients experienced side effects. One of them (Table 1 #9) experienced a slight headache after taking enoximone and another was (Table 1 #10) a mild case of diarrhoea; both issues were solved by distributing the medication over multiple moments of intake/day; they can possibly be traced back to hypersensitivity to the solvents used in enoximone's current liquid form (ethanol and propylene glycol). One other (Table 1 #2) had trouble sleeping and felt restless and agitated. At the time, the patient experienced a burn-out which might also have been responsible for these symptoms. However, this patient did report that she was now, using enoximone, able to inflate a balloon, something she could not do before. All other side effects (see Discussion) have been specifically asked after but were not reported. In contrast, beneficial effects were mentioned: better asthma control, traditional medication being phased down or abandoned, and the disappearance of agitation, tachycardia, and a rushed feeling caused by, e.g., salbutamol or salmeterol when enoximone took their place.

## 4. Discussion

This paper describes as a preliminary report the relevance of PDE3-inhibition, especially enoximone, as a treatment for chronic asthma. Its beneficial effects seem to far exceed its side effects; in addition, patient compliance is high and quality of life is enhanced. Enoximone as an add-on seems to fit in an ICS/LABAs/SABAs reducing regime, and enoximone as stand-alone seems, in many aspects, to be an adequate alternative for the traditional treatments [32]. The fact that even patients from group 1 experience beneficial effects from the add-on therapy (without being able to phase down their traditional medication) is, in itself, an important observation. NB: this group consists of by far the oldest patients, being all women. The aging lung is associated with a decline in lung function and especially in this population represents a more COPD-like endotype with a Th1 and Th17 inflammation profile [33].

A PDE3-selective systemic given drug bypasses particle size and drug inhalator problems and selectively targets exclusively PDE3, even in the very distal small airways [34, 35]. PDE3-expression is mainly limited to the cardiovascular system and lungs and is in general low or absent in other organs. Low-dose PDE3-inhibition provides a quick beneficial airway function-improving effect in severe asthma patients [5–7].

We acknowledge that real-life investigation has its limitations, but it is a meaningful and valuable addition to randomised controlled trials [36]; they each provide equivalent and complementary answers to the same question [37]. This investigation showed that most asthma patients are already familiar with various traditional medications for their condition and can assess quite adequately their need for less/more medication. This need can be influenced by season (pollen, heat, and cold), exercise (sports, games), or incidental illness (flu, common cold). Patient feedback also revealed that time and frequency of intake sometimes made a difference in the efficiency of the working mechanism; 5 mg twice a day, morning and evening, had more effect in some patients than 10 mg once a day. Patients who had contracted a (mostly viral) respiratory infection appeared to benefit from a slightly increased frequency or dosage. For patients who experience a ‘normal' asthma pattern, it does not seem to matter whether the daily dose is 5, 10, 15, or 20 mg. Neither is the severity of asthma a parameter: in most cases, severe uncontrollable asthma responded just as well to 10 mg dd as mild asthma. This indicates a propitious dose-effect ratio; it accentuates that the traditional approach of ‘one dose fits all' does not work in the context of asthma and that drugs should be prescribed based on a personalized dosage, searching for the lowest possible effective dose. In case of exacerbations and additional disorders (colds, respiratory tract infections, pneumonia, and hay fever), an increase in dosage and/or a higher frequency of administration may be applied.

Real-life investigations represent the shift towards personalized medication, based, in this case, primarily on decrease of asthma symptoms and improvement in quality of life [36]. This is also demonstrated from biologicals such as omalizumab (anti-IL-5 in severe asthma); variability in biologic levels has been shown to impact efficacy when also used in other applications of biologicals, e.g., in Inflammatory Bowel Disease [38]: an adapted personalized protocol was needed for more asthma-related benefit [39, 40].

As secondary criteria, substantial reduction of ICS/LABAs/SABAs and high compliance to the drug should be considered. The vast genetic and phenotypical variation of patients suffering from shortness of breath calls for personalized medication. The causes of shortness of breath are multifactorial, involving immune cell inflammation and several structural cells (bronchoconstriction/edema). According to EMA and FDA guidelines, all these cells should be targeted by different drugs, e.g., immune cells by ICS and structural cells by LABAs/SABAs. PDE3-inhibitors target multiple cells at once, including mast cells, basophils, epithelial cells, endothelial cells, smooth muscle cells, and granulocytes. Ideally, for adequate patient compliance, therapies should be easy-to-take and the therapeutic effect should be instantaneously. Our patients distinctly experienced the immediate effects of enoximone treatment (within 10 minutes (median)), which made them more motivated to continue [29, 30, 41], even with the drug in its current liquid formula (requiring additional actions for intake, having an unpleasant taste, and containing a solvent); this underlines the need for adequate medication for (severe) asthma as LABAs/SABAs/ICS apparently are unsatisfactory. In order to eliminate the administration issues, it seems advisable to market enoximone in tablet form; tablets are easy to carry, less socially obvious (in contrast to, e.g., inhalers), and solvent-free and can be taken with plain water, which makes medication compliance much easier. Availability in tablet form will need a complete drug repurposing project.

Existing literature on high dose enoximone mentions several side effects: extrasystoles, supraventricular arrhythmia, ventricular tachyarrhythmia, hypotension, headache, sleeplessness, nausea, vomiting, diarrhoea, mild thrombocytopenia, and a reversible increase of liver enzymes. Rare side effects are cold shivers, oliguria, urine retention, and muscle pain in the extremities [12, 26, 27]. The side effects experienced by the patients in this investigation were of a minor nature and could be solved without affecting the patients' asthma improvement. Two out of three can most probably be attributed to a hypersensitivity to the diluent (propylene glycol, a nontoxic solvent, and antifreeze agent that is often used, in low concentrations, in food (wine), cosmetics, and medicines) in which enoximone is dissolved in its current, liquid form. Beneficial side effects were encouraging: asthma-related comorbidities such as hay fever, allergic rhinitis, allergies, and eczema that appeared to be less pronounced due to enoximone treatment.

The current data show that PDE3-inhibition is profitable not only for acute and very severe asthmatics but also for steroid-using chronic asthmatics [5, 6]. This observation was recently confirmed in a north African study involving the PDE3-inhibitor milrinone, validating both findings on PDE3-inhibition in this paper and in translational research on the beneficial effects of milrinone in allergic airway models [7, 16]. Let it be noted that milrinone has a shorter half-life than enoximone and hence a shorter effect [7, 16]. Milrinone also might represent an exclusion criterion for cardiac patients, as it causes less cardiac diastolic relaxation than enoximone, increasing the chance on arrhythmias [12, 26]. Low-dose enoximone does not have this restriction. Although LABAs/SABAs/ICS have a favorable benefit-to-risk ratio, studies indicate several negative effects; potential adverse systemic effects after long-term exposure include adrenal suppression, decreased bone density, growth suppression, cataracts, yeast/fungal infections, skin alterations, and mood changes, emphasizing the importance of reducing steroids [32]. Of late, more and more data have come available on the use of ICS and beta-2-mimetics being more dangerous than already estimated and that their combined (prolonged) use masks disease severity [3, 4, 42, 43].

As for monoclonal antibody therapy [31], adverse incidents such as exacerbations, hospitalization, autoimmune responses, and autoantibodies have been reported [44]. Monoclonal antibodies are contraindicated in acute asthma exacerbations. In one case, the effect of mepolizumab had worn off after 3 weeks; guidelines allowed the next dose only after 4 weeks. The patient suffered an exacerbation and immediately needed additional supportive medication. He was adequately helped by merely one dose of 15 mg enoximone (personal communication). Dose adaptation should be performed for optimal patient care as reported above for omalizumab [39, 45].

Low-dose PDE3-inhibitors have been administered for long term and prospectively in pediatric heart-related diseases, without mentionable adverse effects, which creates options to use PDE3-inhibitors in pediatric severe asthma [46–48]. Particularly, with children large gains can be achieved regarding the disadvantages of ICS, hospital admissions/complications, and early social stigmatization. Sparing children the inconveniences, disadvantages, and invalidation associated with asthma (and, consequently, avoiding them becoming diseased adults) is obviously essential [28].

Recently, it was observed that intravenous enoximone was able to prevent mechanical ventilation in severe COVID-19 patients via a similar mechanism as seen in near fatal asthma [49]. As severe asthmatics frequently present at the ER, intensive care physicians are usually the first to treat them with enoximone; further implementation of enoximone therapy could be an important step in better asthma control. The ultimate goal might be implementation of the drug at GP level, after having researched the benefit-risk ratio *in extenso*. There still is a large unmet need for effective treatment of severe uncontrollable and chronic asthma; enoximone seems to offer a valid alternative.

### 4.1. Plain Language Summary

Real-life investigational use of the PDE3-inhibitor enoximone in asthma reduces the use of antiasthmatic drugs. Patients clearly experienced the immediate and sustained effects of enoximone treatment by bronchodilation that support therapy compliance and increase quality of life.

### 4.2. Key Messages

PDE3-inhibitor enoximone as (add-on) asthma therapy reduces the need for ICS/LABAs/SABAs/biologicals and hence causes significantly less side effects when compared to ICS/LABAs/SABAs/biologicals.

### 4.3. Capsule Summary

Real-life investigational use of enoximone in asthma of PDE3-inhibitor enoximone is a triple-acting antiasthmatic: it causes bronchodilation, is anti-inflammatory, and works as an antiallergic, suppressing the stimulus that triggers the allergic reaction. It appears to be a valuable alternative for traditional asthma treatment.

## Figures and Tables

**Table 1:** Patients characteristics: age, gender, conventional medication use, enoximone treatment duration, and dosage (Pt = patient; dd = daily dosage; eod = every other day; prn: per as needed).

Pt nr	G**r**oups nr	Age 2019	M/F	First seen	Morbidity	Dosage enoximone	Side effects	Medication before enox.	Medication after enox.	Period of use after starting enox.	Comments
20	1	58	F	March 2019	Asthma	10 mg dd	None	Mometasone 50 mcg nasal spr 1 dd	Idem	>25 months	Was candidate for mepoluzimab; prefers enox.
								Prednisone 30 mg 1 dd Foster aer. 200/6 2 dd	Idem		
								Spiriva resp. 2,5, mcg 1 dd	Idem		
								Foster aer. 200/6 2 dd	Idem		

21	1	54	F	April 2018	Asthma	5 mg dd	Sleeping poorly/restless	Foster 100/6 2 dd	Idem	10 days	Stopped enox. because of side effects
								Beclomethasone 100 prn (nasal spray)	Idem		

27	1	64	F	March 2013	Asthma/hay fever	10 mg 2 x p/w	None	Foster 100/6 2 dd	Idem	>8 years	
								Rhinocort 32 1 dd	Idem		
								Levocetirizine 5 mg 1 dd	Idem		

41	1	76	F	Feb. 2018	Asthma	5 mg dd	None	Salbutamol 100 2 dd	1 dd	>3 years	Appeared to have lung fibrosis, next to asthma. Prednisone for fibrosis could be phased down from 60 to 5 mg dd with help of enox.
								Ciclesonide 80 mcg	Stopped		
								Foster 100/6 2 dd	Stopped		
								After diagnosed with lung fibrosis			
								Prednisone 60 mg dd	5 mg dd		

62	1	40	F	Feb. 2019	Asthma	10 mg dd	None	Qvar 100 2 × 2 dd	Idem	>26 months	

8	2	26	F	June 2013	Asthma/hay fever/	5 mg 2 x dd	None	Ventolin 100 mcg prn	Idem	>8 years	Asthma diminished; hayfever much less; allergies and eczema as good as gone
					Allergies/eczema			Atrovent 20 mcg prn	Stopped		
								Loratadine 10 mg dd	Stopped		
								Xylometazoline prn	Idem		

9	2	38	F	Nov. 2013	Asthma/allergies	15 mg 2 x p/w	None	Ventolin 100 mgc prn	Stopped	>7 years	No more agitation
								Symbicort 200 3 dd	prn		

11	2	60	F	Oct. 2013	Asthma/allergies	15 mg 3 x p/w, extra in case of a cold	None	Airomir 100 5 dd Foster 100/6 4 dd Foradil prn Avamys nasal spr. 2 dd Polaramine 2 mg prn Fexofenadine 180 mg 1 dd Ventolin 200 mcg disc prn	2 dd Stopped Stopped Stopped Stopped Idem Stopped	>7 year	Diagnosed with asthma at very young age; internalized for several years. Severely traumatized by asthma past and always been heavily dependent on meds. Frequent and long-time steroid use caused severe osteoporosis feeling much better with enox.; all steroids stopped! 2015 spirometry best ever!
13	2	43	F	Feb. 2013	Asthma	5 mg dd	None	Salbutamol 100 mcg 12 dd Combivent 6 dd (in conversation 8 x hr) Alvesco 160 2 dd Foradil 2 dd Prednisone Montelukast 110 mg 1 dd	2 dd Idem Idem Idem 5 mg dd Idem	>7 year	Was almost dying - declared untreatable by pulmonologist. On enox. Was able to work and sport again. Stopped enox. And aquired severe stenotrophomonas pneumonia; back on enox. now, with good pulmonary result. Frequent and long-time steroid use caused severe osteoporosis.
14	2	60	M	April 2019	Asthma/hay fever	10 mg dd	None	Ipratropium 250 mcg up to 6 dd ° Salbutamol 100 mcg 1 dd Lorazepam 1 mg 1 dd Foster 100/6 1 dd Seebri 44 mg 1 x dd	Stopped; now Trimbow 2 dd Idem Idem Stopped Stopped	>24 months	
17	2	47	F	July 2018	Hay fever/allergies/eczema	10 mg dd	None	Antihistamine 2 dd Allergodil eye drops prn Steroid cream 1 dd	Stopped Stopped Idem	>30 months	Hay fever significantly better; eczema persistent.
49	2	43	M	Nov. 2018	Asthma	15 mg dd	None	Foster 100/6 4 × 2 dd Seretide 25/125 2 × 2 dd Qvar 100 4 × 2 dd Spiriva 2,5 mcg 1 x dd Rupafin 10 mg prn Livocab 0,5 mg prn Rhinocort 32 1 dd	2 x dd Stopped 2 x dd Idem Idem Idem Idem	>29 months	
54	2	32	M	March 2013	Asthma/hay fever	25 mg 2 x p/w	None	Seretide 25/125 4 dd Fluticasone 100 1 dd Prednisone 5 mg dd	prn Stopped Stopped	>8 years	
55	2	42	F	July 2014	Asthma	5 mg 2-3-x p/w	None	Ventolin 100 mcg 6 dd Seretide 25/125 mcg 3 dd	2 dd 1 dd	>6 years	
56	2	54	M	May 2015	Asthma	10 mg eod (in hay fever season)	None	Foster 200/6 2 × 2 dd Flixonase 50 mcg 2 dd Otrivin nasal spray prn	1 dd 1 dd Idem	>5 years	
63	2	60	F	March 2015	Asthma/allergies/hay fever/Eczema	10 mg dd + enox. cream (4 mg enox./1 ml cream) 1 x dd	None	Flixotide 25/250 mcg 2 × 2 dd Levocetirizine 5 mg 1 dd Montelukast 10 mg 1 dd Ventolin disc 200 mcg dd Elocon eczema cream 1 mg/g 1 dd Prednisone/Prednisolone 5 mg 1 dd	Stopped Stopped Stopped Incidentally/seldom Stopped Stopped	>5 years	Asthma patient ‘since birth' (atopic dermatitis), hay fever, fierce eczema, and allergies to fungal infections due to steroids. Severe traumatization by asthma past. IgE levels extremely high. Steroids all stopped now; asthma, hay fever, eczema and allergies under control. Has common colds less ofte; no more wheezing/expectoration.
2	3	77	F	March 2014	Asthma	15 mg 2 x p/w	None	Alvesco 160 1 dd Foradil 12 mcg 2-4-dd Ventolin disc 4–6 dd	Stopped Stopped Stopped	>7 years	Stopped trad. med. After ca. 4 months
3	3	54	M	April 2015	Hay fever	10 mg dd	None	Several over the counter antihistamines and nasal sprays	Stopped	>6 years	Stopped trad. med. After ca. 2 weeks
4	3	26	M	April 2014	Hay fever	Enox. solution (10 mg/20 ml NaCl 0,9%)	None	Levocetirizine 5 mg prn Xylometazoline 1 mg/ml prn Loratadine 10 mg prn	Stopped Stopped Stopped	>7 years	Stopped trad. med. After 2 months
5	3	41	M	Feb. 2015	Asthma/hay fever	10–15 mg dd	None	Ventolin disc 6-8- dd Seretide 25/125 1 dd	Stopped Stopped	>6 years	Grave cervicothoracic scoliosis; uses BiPAP. Stopped trad. med. After 3 months, now uses 5 mg enox. dd
7	3	66	F	Sept. 2014	Asthma	10 mg dd	None	Spiriva 2,5, mcg 1 dd Seretide 25/125 1 dd	Stopped Stopped	>3 years	Stopped trad. med. After ca. 1 year
12	3	50	F	Aug. 2014	Asthma	10 mg 2 x p/w	None	Ventolin disc 1 dd Spiriva 2,5, mcg 1 dd Montelukast 10 mg 1 dd Alvesco 160 1 dd	Stopped Stopped Stopped Stopped	>3 years	Stopped trad. med. After 6 months
15	3	44	F	July 2016	Asthma/hay fever	5 mg dd	none	Foster 100/6 1 dd	Stopped	>4 years	Stopped trad. med. After ca. 1 year
19	3	51	M	July 2018	Asthma/hay fever/allergies	10 mg dd	none	Nasonex 50 mcg 1 dd Desloratadine 5 mg 1 dd	Stopped Stopped	>30 months	Was even able to stop BiPAP. Stopped trad. med. After ca. 1 year
25	3	63	M	April 2013	Asthma/hay fever/allergies	5 mg dd + enox. cream (enox. 1% in vaseline/vit.E-oil 50/50) prn	Light headache at first; disappeared later	Ventolin 100 mcg prn Combivent prn Loratadine 10 mg 3 dd Dexamethasone cream prn	Stopped Stopped Stopped Stopped;	>8 years	Stopped trad. med. After 2 months
26	3	63	F	April 2018	Asthma	5 mg dd	None	Alvesco 160 2 dd Montelukast 110 mg 1 dd Spiriva 2,5 mcg 1 dd Salbutamol 100 4 dd	1 dd Stopped Stopped prn	>3 years	
28	3	22	M	March 2019	Asthma	5 mg dd	None	Ventolin 100 mcg 6 dd Flixotide 250 mcg 2 dd	Stopped Stopped	>26 months	Stopped trad. med. After ca. 3 months
32	3	68	M	July 2013	Asthma	12,5 mg 2 x p/w	None	Spiriva 2,5, mcg 1 dd Seretide 25/125	Stopped Stopped	>8 years	Stopped trad. med. After 4 months
33	3	45	F	Dec. 2014	Persistent eczema	Enox. cream (enox. 1% in vaseline/vit. E-oil 50/50)	None	Steroid eczema cream 1 dd	Stopped	>7 years	Eczema totally gone. Stopped trad. med. After 2 months
37	3	52	F	March 2019	Hay fever	10 mg prn	None	Aerius 5 mg 2-4-dd Nasonex 50 mcg prn	Stopped Stopped	>26 months	Stopped trad. med. immediately
38	3	45	M	Aug. 2018	Hay fever/allergies	10 mg dd	None	Foster 100/6 2 × 2 dd Montelukast 110 mg prn Ventolin 100 mcg prn	Stopped Stopped Stopped	>30 months	Expects to need enox. Again in hay fever season. Stopped trad. med. After 3 months; stopped enox. After 5 months
43	3	38	M	March 2013	Asthma	10–15 mg 2-3- *x* p/w	None	Ventolin 100 mcg prn Seretide 25/125 2 dd	Stopped Stopped	>8 months	Trad. med. clears up asthma, but when asthma is gone, pruritus in loins comes up - is familial, sister suffers the same. Same happens with enox. Difficult choice.
45	3	18	M	April 2018	Asthma	5–10 mg dd	None	Ventolin 200 mcg 3 dd Avamys 27,5 mcg 2 dd Seretide 50/250 2 dd	Stopped Stopped Stopped	>3 years	Stopped trad. med. After 1 week
46	3	53	M	Sept. 2014	Asthma	10 mg eod prn	None	Ventolin 100 mcg prn	Stopped	>6 years	Stopped trad. med. immediately
48	3	34	M	Aug. 2013	Asthma/allergies	20 mg 2 x p/w	None	Ventolin 200 disc 2 dd Seretide 50/100 2 dd Salbutamol 100 prn	Stopped Stopped Stopped	>7 years	Stopped trad. med. After 6 months
51	3	22	F	Dec. 2013	Asthma	20 mg 2 x p/w	None	Spiriva 2,5, mcg 2 dd 2 puffs Seretide 2 dd 2 puffs	Stopped Stopped	>7 years	Claims that enox. changed her life: is now attending college, does sports and social activities and feels much better in general. Stopped trad. med. After 1 month
57	3	29	M	Oct. 2014	Asthma/allergies	25 mg 1–2 x p/w	None	Ventolin 100 mcg prn Atrovent 20 mcg prn Flixotide 100/6 1 dd	Stopped Stopped Stopped	>6 years	Stopped trad. med. After 1 month
58	3	45	M	March 2014	Hay fever	10 mg eod	None	Several over the counter antihistamines	Stopped	>7 years	Stopped trad. med. immediately
59	3	77	F	Nov. 2013	Hay fever/allergies	5 mg dd	None	Levocetirizine 5 mg dd	Stopped	>7 years	Hay fever/allergies fully under control. Stopped trad. med. immediately
61	3	71	M	Nov. 2013	Asthma	15 mg p/w	None	Spiriva 2,5 mcg 1 dd Seretide 50/250 disc 2 dd 2 puffs	Stopped Stopped	>7 years	Could never function fully; now able to climb stairs, shop, sport (in moderation). Wishes he could have had enox. 40 years ago. Stopped trad. med. After 2 months
6	4	26	F	Feb. 2018	Asthma/hay fever	5 mg dd	None	Flixotide 250 1 dd Ventolin 200 2–4 dd Desloratadine 5 mg 1 dd	Stopped Stopped Stopped	1 year	Stopped trad. med. After 6 months; stopped enox. after 1 year
10	4	44	F	April 2015	Asthma/hay fever	10 mg dd	None	Ventolin 100 mcg 1 dd Pulmicort 100 mcg 1 dd	Stopped Stopped	4 years	Stopped trad. med. After immediately; stopped enox. after 4 years
16	4	58	M	Dec. 2018	Asthma/hay fever	5 mg dd	None	Fostair Nexthaler 100/6 2 dd 2 puffs Flixonase 100 mcg 1-2- dd Pulmicort 200 mcg 2 dd	Stopped Stopped Stopped	1 year	Stopped trad. med. immediately; stopped enox. After ca. 1 year
18	4	35	F	Oct. 2013	Asthma/allergies	10 mg dd	None	Prednisone 20 mcg dd Ventolin 200 2 dd	Stopped Stopped	2 years	Gluten and lactose intolerance as good as gone. Was always reluctant of having children because of fear of passing on her disorders; now has given birth to a healthy child. Stopped trad. med. After ca. 1 year; stopped enox. After ca. 2 years
23	4	50	F	Nov. 2017	Asthma/hay fever	5 mg prn	None	Prevalin 10 mg prn Reactine 10 mg prn	Stopped Stopped	1 year	Stopped trad. med. After ca. 4 months; stopped enox. After ca. 1 year
24	4	70	M	April 2015	Asthma	15 mg eod	None	Ventolin 200 1 dd Spiriva 2,5, mcg 2 dd 2 puffs Salbutamol 100 2 dd 2 puffs	Stopped Stopped Stopped	3.5 year	At 70 yrs old now able to sport 2 x p/w. Stopped trad. med. After ca. 2 months; stopped enox. after ca. 3.5 years
30	4	34	M	Aug. 2013	Asthma	10 mg eod	Mild diarrhoea at first; disappeared later	Pulmicort 200 mcg 2 dd Ventolin 200 mcg 2–8 dd Beclomethasone 5 mg 1 dd Levocetirizine 5 mg 1 dd Natriumcromoglicaat 20 mg/ml prn (eye drops)	Stopped Stopped Stopped Stopped Stopped --	5 years	Farmer - works in dirty, dusty surroundings; yet no more complaints after 5 years of enox. Use. Stopped trad. med. After ca. 2 years; stopped enox. After ca. 5 years
35	4	44	F	Jan. 2014	Asthma	5 mg dd	None	Ventolin 200 mcg prn	Stopped	6 months	Stopped trad. med. immediately; stopped enox. After ca. 6 months
36	4	45	F	Jan. 2015	Hay fever	5 mg dd	None	Levocetirizine 5 mg 1 dd	Stopped	3 years	Stopped trad. med. immediately; stopped enox. After ca. 3 years
40	4	31	M	May 2013	Asthma	25 mg 3 x p/w	None	Seretide 50/100 2 × 2 dd Ventolin 100 mcg up to 10 x dd	Idem Idem	18 months	Stopped trad. med. After 1 week; stopped enox. After ca. 1,5 years
42	4	24	F	Oct. 2014	Asthma/hay fever/eczema	10 mg eod	None	Spiriva 2.5. mcg 1 dd Seretide 25/125 1 dd Ventolin 100 mcg 2–4 dd Steroid cream	Stopped Stopped Stopped Stopped	2 years	Used enox. nasal spray (10 mg enox. in 20 ml NaCl 0,9%). Cleared up hay fever totally. Eczema also gone.Stopped trad. med. After 1 week; stopped enox. After 2 years

**Table 2:** Summary of patients group characteristics including the baselines age, sex, duration of treatment, and the dosage.

Group	Age median range (low high)	Male/female	duration	Dosage mg dd (median range (low high))
A	58 (40–76)	0/5	>2–8 years	5 (3–10)
B	43 (26–60)	4/7	>2–8 years	10 (5–15)
C	48 (18–77)	15/9	>2–8 years	10 (3–15)
D	44 (24–70)	7/4	2 (0,5–6)	5 (5–12,5)

## Data Availability

The data used to support the findings of this study are included within the article.
